# Modeling Electrowetting on Dielectric for Novel Droplet-Based Microactuation

**DOI:** 10.3390/mi15121491

**Published:** 2024-12-13

**Authors:** Behzad Parsi, Max R. Gunn, Jacob V. Winters, Daniel Maynes, Nathan B. Crane

**Affiliations:** Department of Mechanical Engineering, Brigham Young University, 350 Engineering Building, Provo, UT 84602, USA; maxgunn@student.byu.edu (M.R.G.); jwinte42@student.byu.edu (J.V.W.); maynes@byu.edu (D.M.)

**Keywords:** electrowetting on dielectric, micro actuator, microfluidic reconfigurable radio frequency, semi-continuous pump

## Abstract

Recent advancements in Electrowetting on Dielectric (EWOD) systems, such as simplified fabrication, low-voltage actuation, and the development of more reliable materials, are expanding the potential applications of electrowetting actuators. One application of EWOD actuators is in RF devices to enable dynamic reconfiguration and allow real-time adjustments to frequency and bandwidth. In this paper, a method is introduced to actuate a panel using EWOD forces. In the EWOD system, the velocity of the plate increases by maximizing the actuation force, minimizing the moving mass (droplets and metalized plate), and reducing resistance (contact line drag, fluid drag). However, some of these are competing factors. For instance, the actuation force can be increased by increasing the number of droplets, but this also increases the inertia and the drag force. An analytical model of EWOD actuation is presented to understand system performance tradeoffs. The model is validated with an EWOD experiment, and the data demonstrate less than a 7.8% error between the measured and predicted maximum plate velocities for different voltage inputs. In addition, this study presents a 3D numerical FEM model to analyze the velocity profile and viscous force in the thin droplets, focusing on variations along the droplet’s height, which cannot be captured experimentally. The main advantage of the proposed system over previous works is the simple 2D manufacturing process, which allows embedding metalized plates and RF circuit boards, in addition to being compact, portable, and low-cost. In addition, the proposed method does not have any mechanical components, which can increase the system’s reliability in a harsh environment.

## 1. Introduction

Micro actuators are miniature systems that convert various forms of energy into precise mechanical motion at the microscale. They include several types: piezoelectric actuators [1], electrostatic actuators [2], thermal actuators [3], and magnetic actuators [4]. These actuators are important for applications in medical devices, telecommunications, automotive systems, and robotics, offering accurate control in compact designs.

Despite their advantages, like precise control and rapid response [5], micro actuators face several limitations. They generally provide lower forces and displacements and may require high power consumption. The complex and costly fabrication processes, often involving MEMS technology, can restrict scalability. Material limitations, such as the brittleness of piezoelectric materials and the finite durability of shape memory alloys, also present challenges [1,6]. Micro actuators are often large relative to their range of motion, requiring significant space [7]. These microscale systems frequently require more expensive equipment to move parts at slower speeds, compared to their millimeter- and centimeter-scale counterparts [8,9]. Additionally, integrating the micro actuator with other microscale components can be challenging due to alignment issues, and their reliability and lifespan may be compromised by wear and degradation [10].

**Figure 1:**
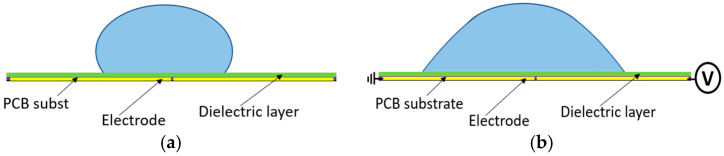
Schematic side-view of the EWOD system (**a**) off, (**b**) on.

At sub-millimeter size scales, fluid movement is largely governed by surface forces, which become dominant. To address some of the drawbacks associated with micro actuators, such as complexity, high fabrication costs, and large size, an Electrowetting on Dielectric (EWOD) driven micro actuator can be utilized. EWOD actuators offer two-dimensional fabrication, which reduces both fabrication costs and complexity. Electrowetting (EW) is a technique, which works by applying electric fields at the liquid/substrate interface, altering the apparent wetting properties and changing the shape of the droplet. This is illustrated in Figure 1 [11], where the drop on the left is the shape that exists prior to the application of an electric field, and the drop on the right represents the change in drop contact angle that can occur.

By modifying the contact angle through electric fields, the movement and positioning of droplets can be controlled [12] with as little as one electrode pair [13]. This ability to control droplet behavior has broad applications in microfluidics, allowing intricate fluid manipulation without the need for traditional mechanical components [11]. This makes EW a versatile approach in scenarios where precision, compactness, and simplicity are crucial, such as in reconfigurable RF devices [14], biomedical applications [15], and lab-on-chip technologies [16]. For instance, Ni et al., in 2016 [12], introduced a new technique for precise droplet positioning using electrowetting actuation, which achieves high-resolution control by varying the duty cycle of an AC signal without the need for closed-loop feedback. The approach enhanced positioning accuracy to within 0.2 mm and repeatability to within 0.07 mm of the droplet diameter.

**Figure 2:**
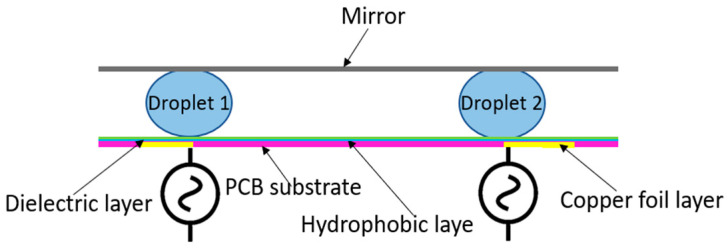
Schematic figure of EWOD actuated optical micromirror [17].

Electrowetting can also be used as an actuator to move or tilt small panels. Kang et al. [17] presented a novel method for controlling the tilting motion of an optical micromirror using EWOD actuation. This idea is illustrated in Figure 2. As voltage potentials are applied to one of the drops, the drop shape changes, and the mirror angle can be adjusted. This technique leverages the vertical displacement of a droplet, allowing simplified micromirror tilting with minimal fabrication complexity and no fatigue of flexures or mechanical wear.

Wan et al. [18] explored the piston motion of a mercury droplet confined in a microscale hole. When the drop was actuated by the electrocapillary effect, displacements up to 210 µm were realized at low voltages (~2 V). They demonstrated a prototype micromirror that could be actuated with a frequency of 400 Hz and an amplitude of about 8 µm, highlighting potential applications in phase-manipulating adaptive optics.

Khodayari et al. [19] showed that valve-metal electrodes, paired with specific electrolytes, can achieve stable operation at low voltages using thin hydrophobic polymer dielectrics. This method, utilizing metals like aluminum, offers a cost-effective solution for electrowetting, with simplified deposition processes that lower commercial production costs. An electrolysis-free electrowetting system has also been introduced that relies on anodic oxide generation, offering complete protection against dielectric breakdown [20]. This system can be easily integrated into existing electrowetting devices by substituting the electrode with an anodizable one and using a suitable electrolyte solution. Another recent project demonstrated the successful development of a simple, low-cost electrowetting lab-on-a-chip using a flexible paper-based design, where droplets can be manipulated with low voltage (12 Vpp) and low frequency (10 Hz) electrical signals [21]. The findings indicate that this new chip can effectively study particle science and engineering. In a separate study, Ni et al. [22] integrated metal-semiconductor diodes into an electrowetting substrate using titanium films, enhancing device reliability and enabling high-speed actuation without degradation after 2000 cycles. This technique simplifies droplet manipulation and could pave the way for servo-style actuation, broadening the scope of electrowetting applications.

Today, most RF devices rely on MEMS technology. Jensen et al. [23] highlighted that while MEMS devices offer benefits, they also encounter challenges such as increased resistance from insulating films and potential reliability issues. These issues can be partially addressed through microfluidic reconfigurable RF devices. However, these microfluidic devices are generally restricted to frequencies below 10 GHz [24,25,26,27,28]. This constraint is primarily due to manufacturing simplicity, RF modeling challenges, and the use of liquid metals. These metals have inherent drawbacks, including lower conductivity compared to traditional materials like copper or gold and reliability issues, such as sticking due to oxidation [24,25,26,27,28].

Mumcu and colleagues [28,29] proposed the concept of selectively metalized plates (SMP), which can move within microfluidic channels. This innovation creates reliable devices with enhanced efficiency and superior power-handling capabilities. In a recent study by Gonzalez-Carvajal et al. [27], a millimeter-wave tunable microfluidic reconfigurable bandpass filter was actuated by pumping fluid between two reservoirs with a piezoelectric disk to move a selectively metallized plate (SMP).

This new SMP technique still relies on micropumps actuators, which are typically embedded within the device or use external micropumps. In either case, these micropumps tend to be relatively bulky. For example, the height of the microfluidic channel walls fabricated by Gheethan et al. [30] was just 203 μm, yet it was actuated by a commercial micropump that is both bulky (39 × 68.5 mm) and costly. González et al. [27] integrated a millimeter-wave hairpin bandpass filter with a PZT micropump. The system’s total dimensions are just 8 mm by 11 mm, thanks to the embedded piezoelectric actuator. Despite the compact and miniaturized design, the manufacturing process involves a cleanroom-based 3D microfabrication technique, which is both costly and time-consuming.

This paper considers the use of an EWOD actuator to actuate a rigid plate that could be used in applications such as an RF device. In the proposed configuration, the plate is supported by four droplets. Actuation forces are applied to the droplets through the activation of electrodes on the substrate. This proposed system utilizes a simple 2D manufacturing process while maintaining compact size, portability, and low cost. Traditionally, electrowetting involves moving droplets to cover an electrode of similar size. However, this proposed system allows the droplet to be shifted in small increments, equivalent to fractions of its diameter, without requiring a closed-loop control system. This precise control makes the EWOD approach ideal for applications in RF tuning and other electronic configurations where accuracy and reliability are essential.

This paper presents a one-dimensional dynamic model of the EWOD system as a function of all geometric and fluid parameters and assesses the estimation of frictional forces on droplets carrying a panel across a hydrophobic surface, considering factors like inertia force, electrowetting force, contact angle hysteresis, and viscous dissipation. Experiments were also conducted, and the experimental results show good agreement with the dynamical model.

## 2. Modeling

**Figure 3:**
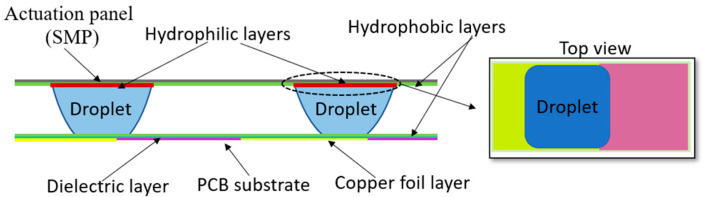
Schematic side-view of the EWOD system.

In this section of the paper, a one-dimensional model of an EWOD actuation setup that can move the SMP (actuation panel) is developed. The system is shown in Figure 3. Electrodes exist in the PCB substrate and are covered by a dielectric layer. On top of the dielectric layer, a hydrophobic layer is placed. Droplets are placed into confined positions and the actuation panel rests on top of the droplets.

The EW actuation relies on a grounded droplet configuration [31], where the droplet is maintained at ground potential, typically with a grounded top plate or a thin wire. When a voltage is applied to a dielectric-covered electrode, the contact angle for the drop over that electrode suddenly decreases. The droplet then moves until it covers the energized electrode. The electrodes are positioned along the bottom surface while the moving plate rests on the drops and moves with them. To maintain continuous actuation, voltage is applied successively to each electrode in front of the droplet, propelling it forward, while the electrodes behind the droplet are grounded. This process maintains a semi-continuous electrowetting force and can enable precise positioning and consistent operation in applications requiring a reliable actuation method. The droplet voltage is maintained in the ground state by one or more wires on the substrate.

The objective of the analysis is to develop a simple predictive model that will allow exploring the influence of all of the system variables. All properties of the liquid droplet and surrounding fluid are assumed to remain constant. Applying a force balance that includes electrowetting, contact angle hysteresis, contact line friction [32], and viscous dissipation forces. In addition to the drop inertia yields the following expression:

(1) $$F_{ew}-\alpha A\mu_{f}\bar{u}-\eta A\bar{u}=(\rho_{d}\forall_{d}+\rho_{f}\forall_{f})\frac{d\bar{u}}{dt}$$

where $\forall_{d}$ and $\forall_{f}$ and ρ_d_ and ρ_f_ are the volumes and densities of the droplet and surrounding fluid, respectively, and A is the area of the hydrophobic region. $\bar{u}$ is the instantaneous average velocity of both the droplet and surrounding fluid, and μ_f_ is the viscosity of the drop. α is a parameter that accounts for the droplet viscous effect and η is the contact-line friction coefficient (molecular friction factor). Both α and η are parameters that need to be evaluated experimentally, and how this is conducted is explained in Section 4.

The electrowetting force ${(F}_{ew})$ is determined by the energy gained from displacing the contact line. The electrowetting force of a 2D droplet can be calculated as [19]:

(2) $$F_{Ew}=l\gamma_{LG}\left[\left(\cos\left(\theta_{1}\left(v\right)\right)-\cos\left(\theta_{3}\right)\right)+\left(\cos\left(\theta_{4}\right)-\cos\left(\theta_{2}\right)\right)\right]$$

where $\gamma_{LG}$ is the surface tension coefficient between the droplet and the surrounding fluid, l is the width of the droplet, $\theta_{1}$, and $\theta_{3}$ are the advancing and receding apparent contact angles of the bottom part of the droplet over the electrode, respectively, and $\theta_{4}$, and $\theta_{2}$ are the advancing and receding apparent contact angles of the droplet.

## 3. Experimental Methods

The fabrication and assembly process, along with a comprehensive analysis of the electrowetting response is provided below. Additionally, the experimental setup, including the specific equipment and methodologies used to record data, is described.

### 3.1. Fabrication Process of the Panel and Prototype

The panel to be actuated by the droplets was fabricated using the vat polymerization method. The panel provided hydrophilic regions for the droplets that were surrounded by the hydrophobic areas to define the droplet-wetting region and support accurate motion of the plate in response to droplet actuation. As Ramadoss et al. [33] demonstrated, since the droplet is thin, the force equation assumes that the contact area between the fluid and the substrate closely matches the area of the plate, maintaining accuracy even under slight misalignments or variations in z-height.

**Figure 4:**
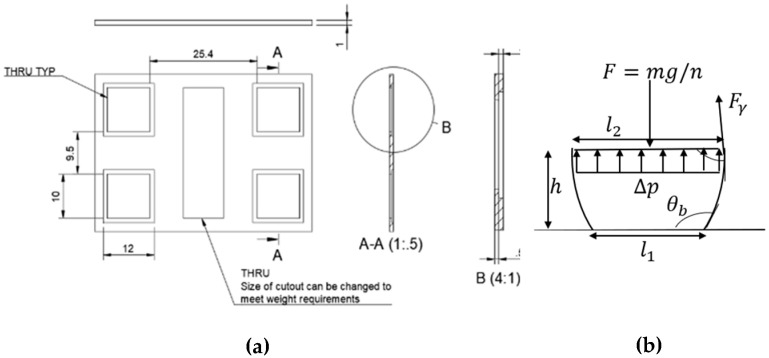
(**a**) Dimensions of actuation panel (“THRU TYP” stands for “Through Typical”). (**b**) The force balance of one of the n droplets.

**Figure 5:**
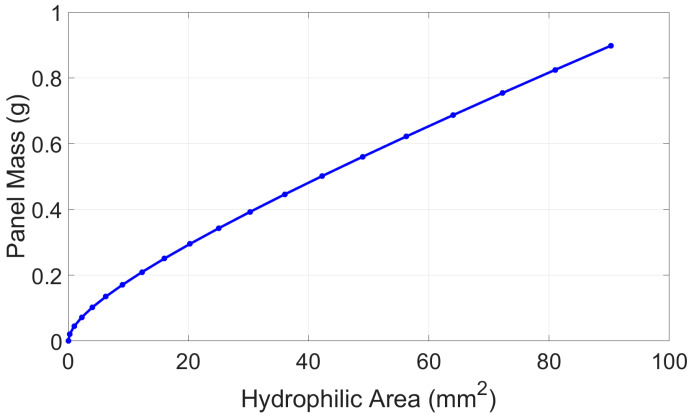
Estimation of the maximum allowable panel mass as a function of the required hydrophilic area.

Figure 4 illustrates the force balance of one of the four droplets. As shown, the panel’s weight (m) must be balanced by the internal Laplace pressure and surface tension forces. In the design, the maximum calculated weight of the panel that could be stabilized by the four droplets is 0.92 g. In the prototype, the droplet was sandwiched between a hydrophilic and a hydrophobic surface, making it challenging to analytically determine the exact center of curvature. Therefore, an estimation of the principal curvature was used to calculate the weight with a high safety factor, meaning the actual load-bearing capacity may exceed the calculated value. However, in cases where the droplet is between two hydrophobic surfaces or inside a channel with a known height, the analytical model can precisely predict the radius of curvature. For example, Ni et al. [34] demonstrated an analytical model for droplets between two wetting surfaces with a known height, accurately predicting the droplet’s curvature and its influence on stiffness. The equation for mass estimation can be defined as follows:

(3) $$m=\frac{n}{g}\left(F_{\gamma }\sin\left(\theta_{t}\right)+∆pA\right)$$

where n is the number of droplets, $F_{\gamma }=\gamma \times perimeter$, and $∆p=\gamma \left(\frac{1}{R_{1}}+\frac{1}{R_{2}}\right),\;R_{1}\approx \frac{l_{1}}{2}=\frac{l_{2}}{2}=$ 5 mm, $R_{2}=\infty$. In addition, in Figure 4, h is the height of the droplet, $l_{1}$ is the non-wetting length, and $l_{2}$ is the wetting length. Figure 5 shows the plot of Equation (3) as a function of the required hydrophilic area.

In designing the panel, the width and length were fixed due to the constraints imposed by the electrode fabrication size, leaving the panel’s height as the only variable (See Figure 4a,b). However, due to the limitations of the SLA printer, the panel thickness could not be reduced to below 500 μm. To achieve the desired weight, material was removed from the center panel, as illustrated in Figure 4a,b. The hydrophilic droplet regions were created by attaching hydrophilic coverslips (12×12 mm) to the panel, while the remaining areas were coated with a hydrophobic layer. The hydrophobic layer coating method is discussed in Section 3.2.

**Figure 6:**
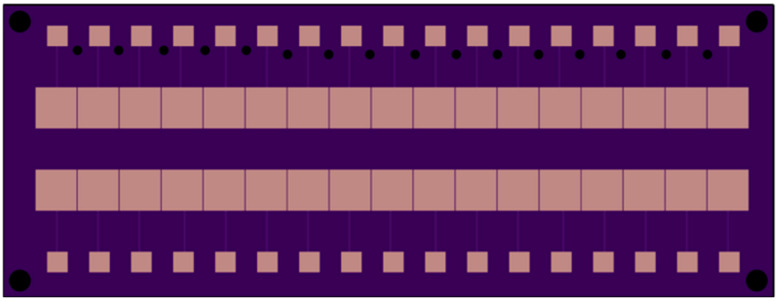
Electrodes pattern.

The substrate (Figure 6) is a print circuit board (PCB) over bare copper (SMOBC) and an Electroless Nickel Immersion Gold (ENIG) finish from OshPark. The EWOD preparation steps, such as covering the PCB with dielectric layer and hydrophobic layer, are discussed in Section 3.2.

### 3.2. EWOD Preparation and Characterization

This subsection details the preparation of electrodes and the application of coating layers for optimal electrowetting performance. Initially, the electrodes on the PCB were covered with a 100 μm layer of acrylic packing tape to serve as a dielectric. A commercial multi-surface Neverwet spray was then applied as a hydrophobic layer to reduce wetting hysteresis [35]. The base coat ensured adhesion to the surface and imparted a rough texture, while the topcoat created a water-repellent layer. It is noted that the effectiveness of the Neverwet coating can diminish over time [35]. Consequently, both the dielectric and hydrophobic layers were replaced before each test.

The electrowetting contact angle response was measured on an exposed electrode to estimate the electrowetting forces and determine the saturation voltage. A grounded electrode setup was employed, with an AC voltage (frequency = 1 kHz) applied to the droplet.

**Figure 7:**
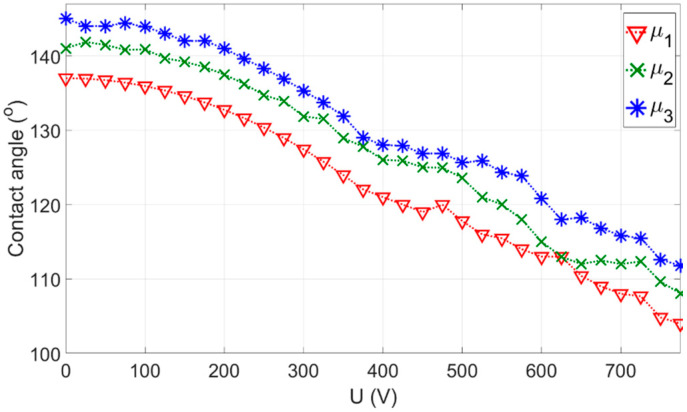
Measured static contact angle of the droplet on the coated dielectric layer as a function of applied potential (EWOD curve) for different fluid viscosity: μ1 =0.001 kgm·s, μ2=0.002 kgm·s, and μ3=0.003 kgm·s.

A DI water droplet with a volume of 15.7 µL was deposited on the grounded electrode using an automatic syringe pump, with a grounded wire positioned at the droplet’s center. The droplet’s shape was imaged as a function of the applied voltage, and the contact angle was determined using the plugin Contact Angle of ImageJ software. All measurements were taken under ambient atmospheric conditions at room temperature. As depicted in Figure 7, the contact angle is at its maximum (137.1°) for fluid 1 when no voltage is applied. In the Young-Lippmann (YL) regime, the contact angle decreased progressively and at an increasing rate with increasing voltage. Figure 7 shows the static contact angle of the droplet as a function of the applied voltage, U. The data demonstrate the Young-Lippmann regime for U < 750 V, while for U > 750 V, the electrowetting response saturates and the contact angle stabilizes at a constant value. The solid line in Figure 7 is a fit of the YL equation to the experimental data, where the insulator thickness d and $\varepsilon_{r}$ was taken as a fit parameter, and can be defined as $\alpha =\frac{\varepsilon_{o}\varepsilon_{r}}{2\gamma d}=(\cos\theta \left(U\right)-\cos\theta_{o})/U^{2}=7.57\times 10^{-7}(\frac{1}{V^{2}})$.

### 3.3. Experimental Procedures

The primary objective of the experiments was to investigate the influence of the actuation force on the velocity of the panel. First, the velocity of the panel was measured as it moved across the PCB electrodes under the influence of a step input to one or more electrodes. This test was conducted with different electrode potentials and the time response of the panel was compared to the model, as described by Equation (1). Secondly, the resistance force was investigated by varying the viscosity of the system (μ1,μ2,μ3) by adding glycerol to water to increase the viscosity of the resulting mixture. Additionally, the impact of changing the width of the electrodes was examined to assess the accuracy of the model as the actuation force increased.

**Figure 8:**
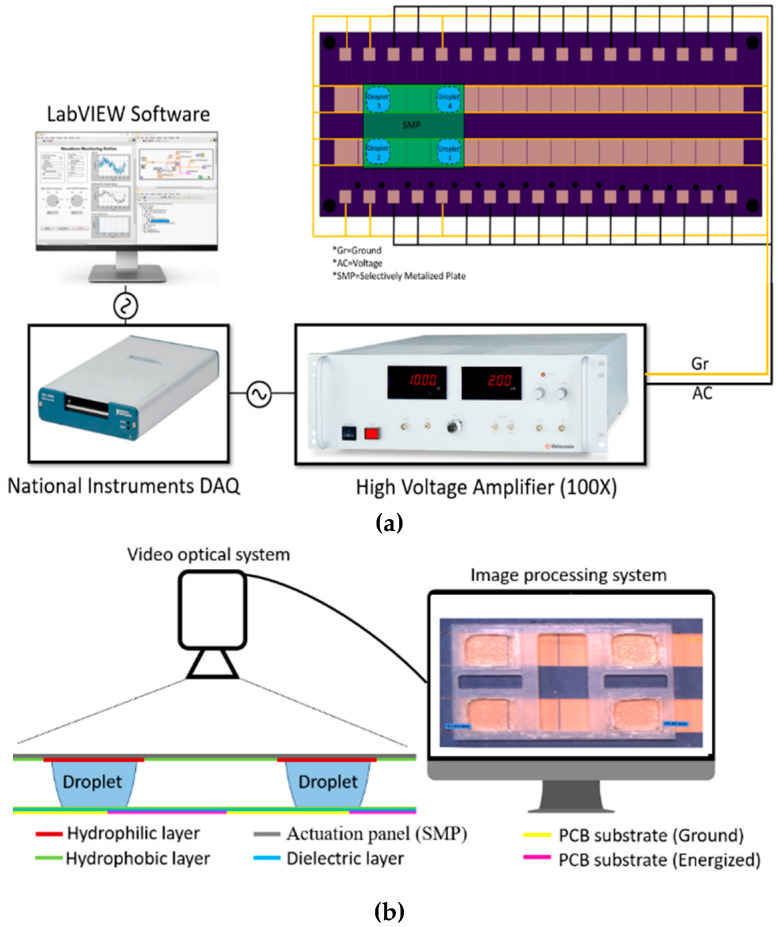
(**a**) The setup electrical test. In this setup, an AC voltage with an amplitude ranging between 0 and 10 V was generated at a frequency of 1 kHz using an NI USB-6343 signal generator and it is then amplified 100 times using a high voltage amplifier (Matsusada-COR-10B2). This voltage was then applied to the PCB to actuate the droplet motion. (**b**) Side view of the setup and electrodes with optical droplet measurement technique.

The experiment was initiated by depositing four drops of the fluid of interest with a volume of 250 µL over the ground electrode using an automatic syringe pump. The experiments were conducted in ambient air at a temperature of 20 °C and a relative humidity of 68%. The electrical setup is depicted in Figure 8a. An AC voltage with variable amplitude from 0 to 10 V was generated at a frequency of 1 kHz using the NI USB-6343 signal generator. Subsequently, the AC voltage was amplified 100-fold using the Matsusada-COR-10B2 high-voltage amplifier. The applied voltage altered the apparent contact angle between the solid surface and the fluid to actuate the droplet. To obtain velocity and acceleration from high-speed camera footage using MATLAB, the droplet’s position was tracked in each frame, and velocity was computed as the change in position divided by the change in time. Subsequently, acceleration was calculated as the change in velocity divided by the change in time. Each test was repeated four times, and the average velocity across the four tests is plotted. The experiment parameters are shown in Table 1.

### 3.4. FEM Model

A 3D numerical FEM model of the droplet was created to study the velocity profile and viscous dissipation force along the droplet’s height, focusing on the differences between the middle and the edges of the droplet which cannot be observed experimentally. In Section 2, the viscous dissipation force, F_f_ = αAμ_f_$\bar{u}$, where α represents the droplet’s viscous effects and dimensional coefficients, was introduced. The exact value of α can be determined by fitting Equation (1) to experimental data. However, the numerical FEM model provides insight into how the droplet’s height, length, and width affect the value of α.

In this paper, 3D numerical simulations were carried out using COMSOL™ Multiphysics software version 5.6. The Phase Field method was employed to model the fluid–fluid interface between the droplet and the surrounding environment. This approach is particularly useful for capturing the multiphase flow dynamics inherent to the proposed setup [36]. The Phase Field method yields highly accurate results in shorter computational times. This efficiency becomes especially clear when compared to level set methods [37,38].

**Figure 9:**
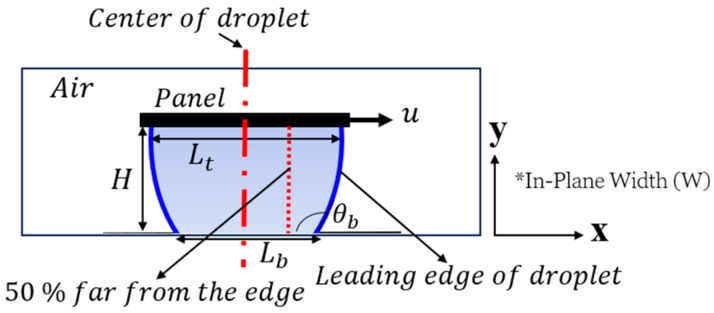
Schematic side view of the 3D FEM, where the in-plane length is denoted as W, details regarding the test parameters can be found in Table 1.

The 3D simulation began with all fluids initially at rest. The top and bottom walls were specified as no-slip boundaries (see Figure 9) and the panel moved with a steady state velocity (u = 0.03 m/s); other details regarding the test parameters can be found in Table 1. The outlet was defined as the exit point for the domain to the atmosphere pressure. In the Phase Field module, the parameter $\varepsilon_{pf}$, measured in meters, governs the interface thickness within a model. By default, it is set to half the size of the largest mesh element in the interface region. The mobility tuning parameter, χ, with units of meters per second per kilogram, affects the time scale of Cahn–Hilliard diffusion, thereby influencing the rate of diffusion at the interface. This parameter was manually defined, typically defaulting to 1 ms/kg, which is suitable for most models. Additionally, an adaptive mesh algorithm was used to dynamically adjust the finite element mesh along the liquid–gas interface every 2 ms. The convergence was achieved when the residuals of continuity, momentum, and energy equations fell below $10^{-6}$, with velocity fields showing changes of less than 0.1% between iterations. A grid-independence study, conducted with normal and ultra fine elements demonstrated less than 0.1% variation in key parameters, confirming the solution’s independence from mesh size and the best mesh size was selected.

## 4. Results and Discussion

**Figure 10:**
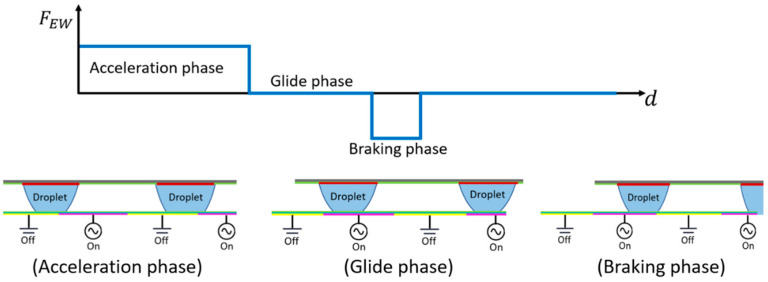
Illustration of the droplet motion as subsequent electrodes are activated; in this setup, when the electrode is activated (ON), the droplet is propelled forward by the electrowetting force. However, when the droplet reaches the grounded electrode, the electrowetting force diminishes to zero.

To verify the proposed analytical model (Section 2), the velocity response of the panel under applied voltages was measured experimentally. When voltage was initially applied (acceleration phase), the droplet was propelled forward by the electrowetting force. However, when the droplet completely covered the active electrode, the electrowetting force diminished to zero (glide phase), and the droplet began to decelerate due to the friction force (see Figure 10). If the droplet reaches a grounded (zero voltage) electrode, the actuation force reverses, and deceleration increases significantly (braking phase).

### 4.1. Estimation of Viscous Effect and Contact-Line Friction Coefficient

This section calculates the exact values of the droplet viscous coefficient (α), and the contact-line friction coefficient (η) by fitting Equation (1) to the experimental data.

**Figure 11:**
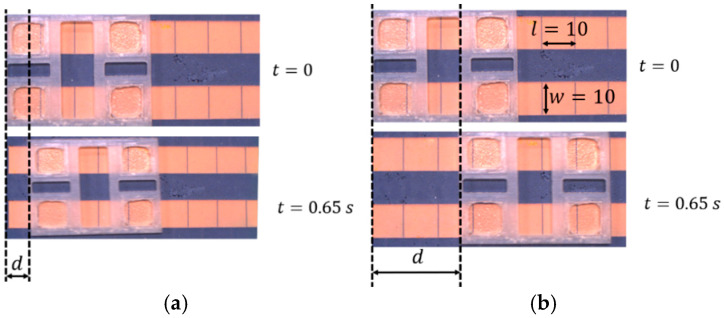
Illustration of the droplet motion as subsequent electrodes are activated; in this setup, when the electrode is activated (ON), the droplet is propelled forward by the electrowetting force. (**a**) v = 273 V, (**b**) v = 707 V.

Figure 11 represents the motion of the panel across the hydrophobic surface for two different applied voltages. In these two tests, all parameters from the methods section and Table 1, including droplet volumes, panel dimensions, and test conditions, were kept constant while the electrowetting force increased by raising the actuation voltage from 273 V to 707 V. As a result, the actuation force increased while the inertial force remained constant, leading to faster acceleration and greater distance traveled by the panel.

**Figure 12:**
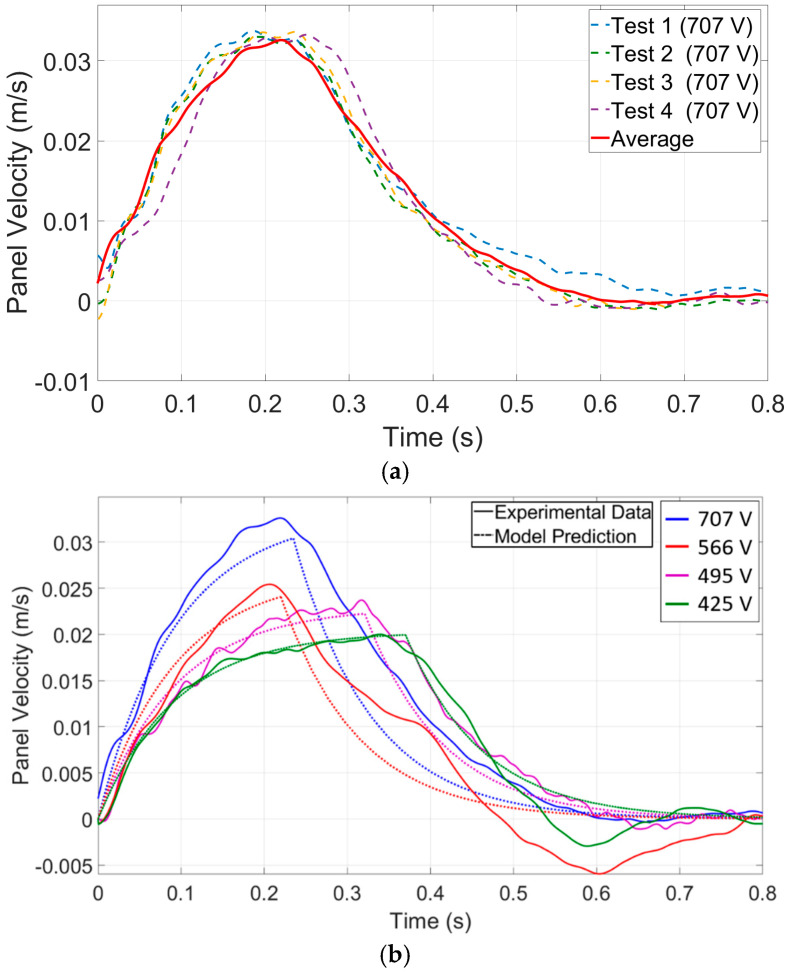
(**a**) The measured panel velocity as a function of time for one applied voltage and four tests in addition to the average of these tests. (**b**) Analytical model and measured droplet velocities as a function of time for four applied voltages. Details regarding the test parameters can be found in Table 1, while the test procedures are elaborated in the method section.

In the experiment, each test was repeated four times. Figure 12a shows the panel velocity as a function of time for each replication of a test, along with the average velocity. As observed, the average value provides a reliable representation of the system’s response. Figure 12b and subsequent figures plot only the average of each test condition.

Section 2 presented an analytical model to describe the system’s dynamics. However, two constants, α (viscous effect coefficient) and η (contact-line friction coefficient), must be determined empirically through experimentation by fitting Equation (1) to the experimental data. To generate these data, the experimental setup, as described in the methods section, measured the panel velocity as a function of time under different actuation voltages. Using these data, the non-linear least-squares optimization function from MATLAB R2020b was employed to minimize the difference between the experimental data and model predictions by adjusting parameters: the droplet viscous coefficient (α), and the contact-line friction coefficient (η). This method defines an objective function based on residuals $\left(f=F_{ew}-\alpha A\mu_{f}\bar{u}-\eta A\bar{u}-\left(\rho_{d}\forall_{d}+\rho_{f}\forall_{f}\right)\frac{d\bar{u}}{dt}\right)$. Table 2 summarizes the extracted numerical coefficients.

Figure 12b compares experimental data and model predictions of panel velocity as a function of time under different actuation voltages. It is evident that the model generally captures the overall trend of the panel velocity, with both the experimental and predicted data showing a rapid increase to a peak followed by a gradual decline. However, some discrepancies can be observed, particularly in the magnitude of the peak velocities and the timing of these peaks. For instance, at higher voltages (707 V and 637 V), the model predictions closely align with the experimental data, although slight underestimations are noted. In contrast, at lower voltages (566 V and 496 V), the model seems to predict the maximum velocity more accurately than at higher voltages. Currently, the maximum error between the peak velocity of the panel in the experiments and the model is 7.8%, which can be reduced by limiting the working voltage range.

Another source of error arises from the assumption of a square droplet shape used throughout the analysis. This assumption was applied to calculate the gap height based on the droplet volume and to determine the wetted area of the substrate. In reality, the droplet had curved surfaces, leading to inaccuracies in these assumed values. Additionally, the current test setup operates under open atmospheric environment conditions, with each test repeated four times while droplets are exposed to ambient air. Droplet evaporation during testing could introduce errors in droplet volume and mass calculations. For future considerations, integrating the system with an RF device to create a closed-form system could mitigate the evaporation issue.

The following subsection examines factors influencing actuation speed, including friction forces, electrowetting force, and number of droplets.

**Figure 13:**
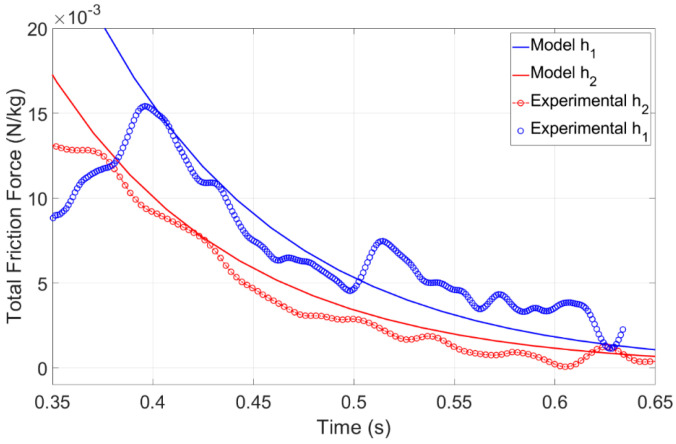
Comparison between the model’s total friction force, accounting for the internal viscous friction within the droplet ($f_{vis}$) and contact line friction ($f_{CL}$), and the experimental data’s total friction force, in deceleration phase for two different heights $h_{1}=0.95\;mm$ and $h_{2}=1.24\;mm$.

To gain more insights into the friction coefficient, studying the total force during the deceleration phase is beneficial. The total friction force can be calculated by taking the derivative of the velocity profile during this phase, where the electrowetting force diminishes to zero. However, calculating the derivative of velocity can introduce noise into the system, making it difficult to fully compare the experimental data with the analytical model, as shown in Figure 13. Nonetheless, this plot reaffirms that the viscous friction coefficient changes with the height of the droplet, as described in the previous section. Specifically, as the height increases, the viscous friction force decreases. Further studies are required to measure the friction force using a force sensor.

### 4.2. Effect of Viscosity on Actuation Speed

**Figure 14:**
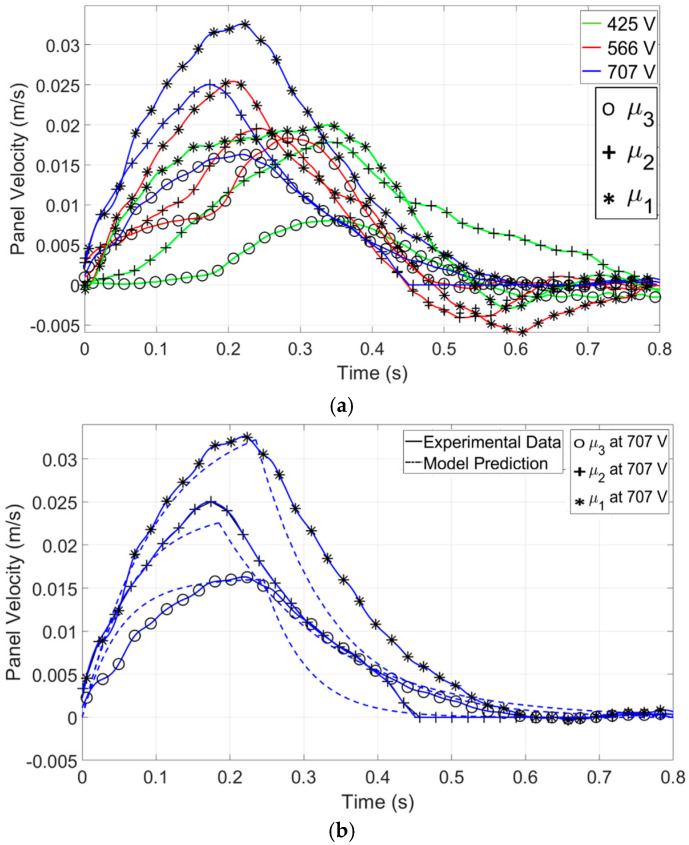
(**a**) Experimental data of droplet velocities as a function of time for three applied voltages while the viscosity changes from μ1 =0.001 kgm·s to μ3=0.003 kgm·s. (**b**) Comparison between the model prediction and measured droplet velocities from the experiment as a function of time for an applied voltage while the viscosity changes from μ1 =0.001 kgm·s to μ3=0.003 kgm·s. Details regarding the test parameters can be found in Table 1, while the test procedures are elaborated in the method section.

To verify the effect of viscosity on the model, the same experimental setup, as introduced in Section 3.3, and the same constants, α (viscous effect coefficients) and η (contact-line friction coefficient), as listed in Table 2, were used with different droplet viscosities. Figure 14a demonstrates the impact of electrowetting and viscous forces on the velocity of a panel over time. Electrowetting force, controlled by the applied voltage (425 V, 566 V, and 707 V), is responsible for the initial acceleration of the droplet. Higher voltages increase the electrowetting force, leading to higher peak velocities. On the other hand, the viscous force, determined by the viscosity of the liquid (μ1=0.001 kgm·s, μ₂=0.002 kgm·s, and μ₃=0.003 kgm·s), opposes the motion of the droplet. Higher viscosities (μ₃) result in greater resistance to flow, leading to lower peak velocities and faster decay in velocity after the peak.

Figure 14b compares the panel velocity over time for droplets moved by EWOD force at 707 V, contrasting experimental data with model predictions for three different viscosities ($\mu_{1},\mu_{2},\mu_{3}$). The model generally follows the experimental trend but underestimates the peak velocity and overestimates the time to reach it. Surface heterogeneities, such as roughness, chemical contamination, or defects, can significantly affect droplet behavior during electrowetting actuation. In reality, surfaces often exhibit microscopic roughness or defects that can trap the contact line, hindering smooth motion [39]. This can lead to slower droplet movement and discrepancies between experimental results and model predictions. For higher-viscosity fluids, the impact of these surface heterogeneities is even more pronounced, as the viscous forces resist deformation [40]. As shown in the contact angle-voltage plot in the methods section, the contact angle does not consistently change with increasing voltage, particularly in regions where viscosity is high. This suggests that the droplet may be sticking to the surface. The increase in viscosity likely leads to enhanced contact line pinning, where the edge of the droplet becomes stuck to the surface, preventing smooth movement, especially during the acceleration phase. Consequently, the droplet exhibits shape asymmetry, with one side displaying a higher contact angle than the other.

Furthermore, the increased viscosity contributes to greater contact angle hysteresis, which negatively impacts the droplet’s mobility. This heightened hysteresis means that more force is required to move the droplet, resulting in stickier behavior and further contributing to the observed asymmetry.

### 4.3. Effect of Electrode Size on Actuation Speed

**Figure 15:**
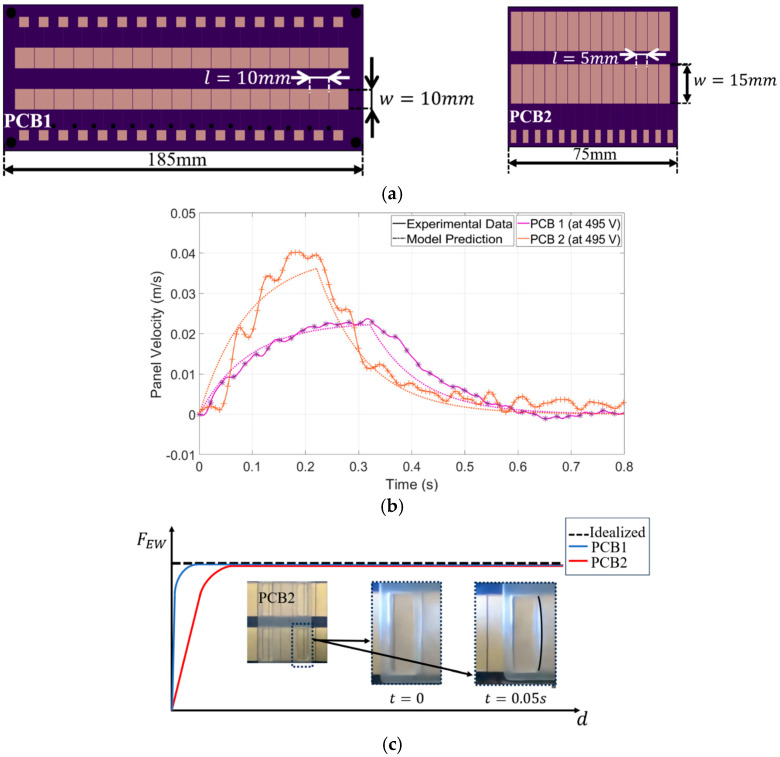
(**a**) Electrodes patterns for two different PCB designs; (**b**) the idealized and ramped electrowetting force for wider droplet (PCB2); (**c**) comparison of panel velocity driven by electrowetting force for PCB1 (10 mm width) and PCB2 (15 mm width) at 495 V.

This section compares two PCB shapes designed for actuation (see Figure 15a), highlighting the differences in their electrowetting forces and resulting performance. Between the two, PCB2, which has a wider shape and generates a larger electrowetting force than PCB1, enabling faster actuation. Experimental data are then analyzed to assess the accuracy of theoretical predictions. Figure 15b compares the performance of the two PCBs designed to move the actuation panel at 495 V. The redesign of the PCB, reducing droplet volume by 14% and increasing panel thickness by 24%, maintains similar inertia forces. PCB2, which is wider (w= 15 mm vs. *w* = 10 mm in PCB1), generates a higher electrowetting force, resulting in faster actuation.

Although theoretically, the inertia force is considered the same for PCB1 and PCB2 in both experiments, the preparation of PCB2 and the addition of droplets are much more challenging, which may contribute to inaccuracies in droplet volumes and errors between theoretical predictions and experimental results. Another source of error arises from assuming a rectangular droplet shape throughout the analysis, this assumption was used to calculate the gap height based on droplet volume and to determine the wetted area of the substrate, but in reality, the droplet had curved surfaces, leading to inaccuracies. In Figure 15c, the initial discrepancy is likely due to the curved contact line of the droplet’s leading edge in PCB 2 (L/w = 0.33), which results in a gradual electrowetting force when the voltage was applied (see Figure 15b).

In this study, the droplet height is approximately 1.2 mm due to experimental challenges associated with smaller droplets and panel sizes. However, smoother velocity profiles could be achieved with smaller heights, as the electrowetting force would act more spontaneously rather than exhibit a curved behavior. As demonstrated by Ramadoss et al. [33], the contact area between the thin droplet and substrate closely matches the plate’s area for smaller droplet heights, ensuring accuracy despite slight misalignments.

### 4.4. Viscous Friction Force Study by FEM

To better understand the sources of error between the analytical model and experiment, a 3D numerical FEM model of the droplet was developed as described in the method section to analyze the velocity profile and viscous dissipation forces along the droplet’s height, with a focus on the differences between the center and edges of the droplet that are not observable in experiments.

**Figure 16:**
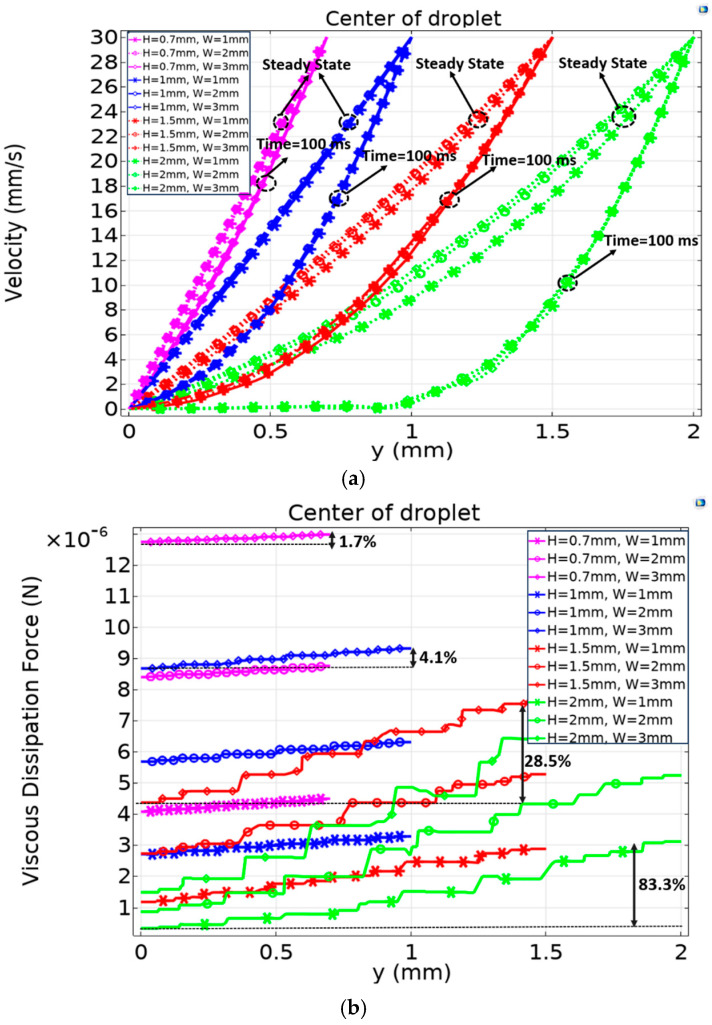
(**a**) Droplet fluid velocity profile as a function of a gap under different maximum height and width configurations. (**b**) Viscous dissipation force of droplet as a function of gap under different maximum height and width configurations. The ‘y’ coordinate represents the distance from the substrate.

Figure 16a presents the 3D numerical FEM simulations results, utilizing the Phase Field method, and demonstrates the impact of droplet dimensions on velocity profiles. Smaller droplets, with reduced height and width, exhibit faster response times and maintain behavior closer to linear. In contrast, larger droplets show slower velocity growth and take longer to reach a non-linear steady state. In all cases, the steady-state profile is non-linear and not a perfect line. Additionally, due to the jerky behavior of real droplets in electrowetting actuation, the droplet never fully reaches a true steady-state phase, so the transient phase in Figure 16a has an important role in the electrowetting system.

This figure also shows that the width of the droplet does not affect the velocity profile at the center of the plate as much as the height does, with this effect approaching zero for a height less than 1 mm. Therefore, it is helpful to choose parameters that maintain linearity in the system. The simulation shows that if the droplet’s height-to-width ratio is below 0.05 (H/W = 0.05) the system’s behavior is highly linear and, thus, more predictable.

Figure 16b shows the viscous dissipation force. In a purely linear scenario, the viscous dissipation force would form a completely horizontal line. However, Figure 11b confirms the non-linear behavior observed in Figure 16a. Smaller droplets have higher dissipation forces, but Figure 16a (with H/W = 0.05) shows that the thinner droplets (small H), maintain a relatively constant dissipation force, while taller (large ‘H’) droplets exhibit a marked increase in dissipation along the gap. This emphasizes the importance of controlling the gap size to develop an analytical model (as in Equation (1)) that can accurately predict the system’s behavior based on experimental data.

**Figure 17:**
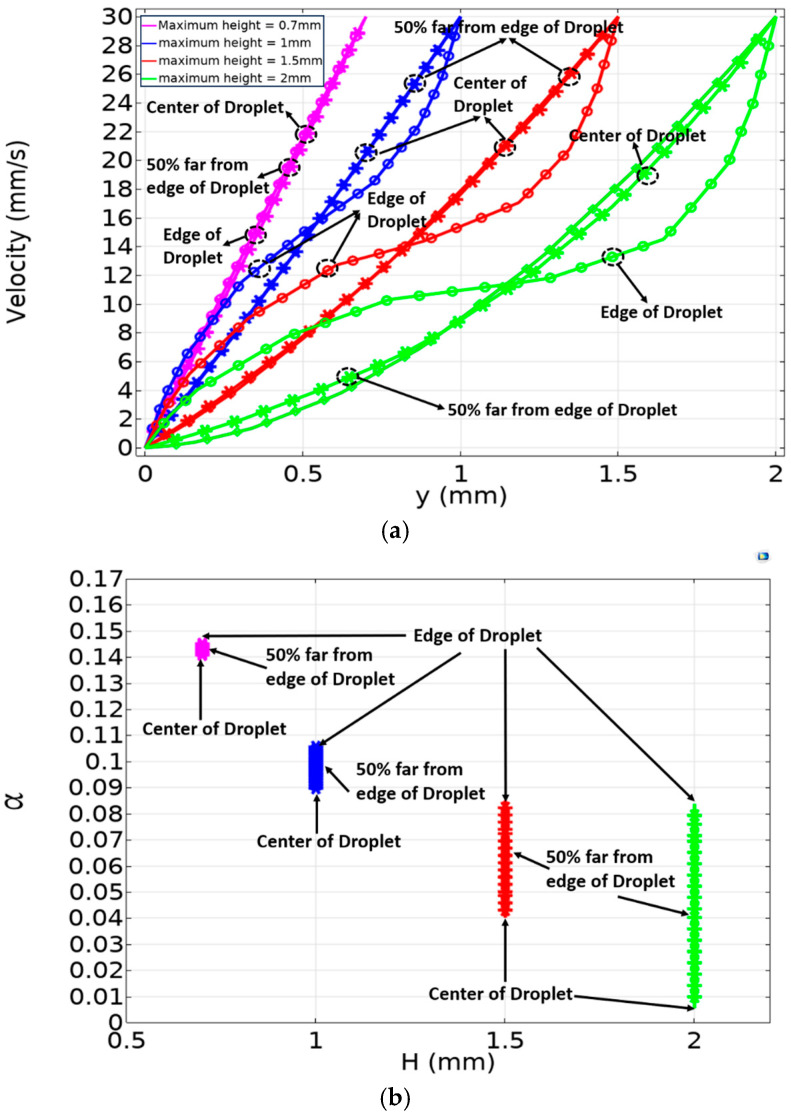
(**a**) Velocity profile of droplet as a function of gap for different maximum heights of a droplet (0.7 mm, 1 mm, 1.5 mm, and 2 mm), where the data points represent velocities at the center of the droplet, 50% from the leading edge, and at the leading edge of the droplet at t = 100 ms. (**b**) Coefficient α as a function of droplet height for three different regions: center, 50% far from the edge, and at the edge of the droplet.

The 3D numerical FEM simulations provide a more detailed understanding than experimental methods. Figure 17a shows the velocity profile as a function of gap for three different locations: the center of the droplet, 50% far from the leading edge, and at the leading edge of the droplet. Due to the stick-slip behavior of large droplets tested in electrowetting actuation, as discussed above, the droplet may never fully reach the steady-state phase, so the transient phase at (t = 100 ms) was plotted, as shown in Figure 17. In Section 2, the analytical model (Equation (1)) assumes that the velocity profile is uniform across the droplet, but the detailed numerical FEM study reveals that the velocity varies across the droplet. This variation in terms of viscous friction coefficient, (α), is clearly shown in Figure 17b.

From this variation, it can be observed that the viscous friction coefficient, (α), changes by 800% at (H = 2 mm) from the leading edge to the center of the droplet, while the variation is much smaller (4.5%) at (H = 0.7 mm) from the leading edge to the center of the droplet, showing an almost smooth gradient across the droplet and behavior closer to the analytical model. This highlights again the importance of controlling the gap size to develop an analytical model (as in Equation (1)) that can accurately predict the system’s behavior based on experimental data.

### 4.5. Other Design Considerations

The model can be used to assess the effects of droplet configurations on actuation speed, particularly in scenarios where experimental validation is difficult, such as nanoliter droplet generation or precise droplet spacing. It helps evaluate the performance of the proposed EWOD actuator and identify conditions that could enhance its competitiveness as an actuation system. Two case studies were conducted, using simulation parameters from Table 1. The electrical setup closely followed the methods section, with the key difference being that additional electrodes were actuated at precise times to maintain a constant electrowetting force on the plate, preventing it from entering a deceleration phase.

**Figure 18:**
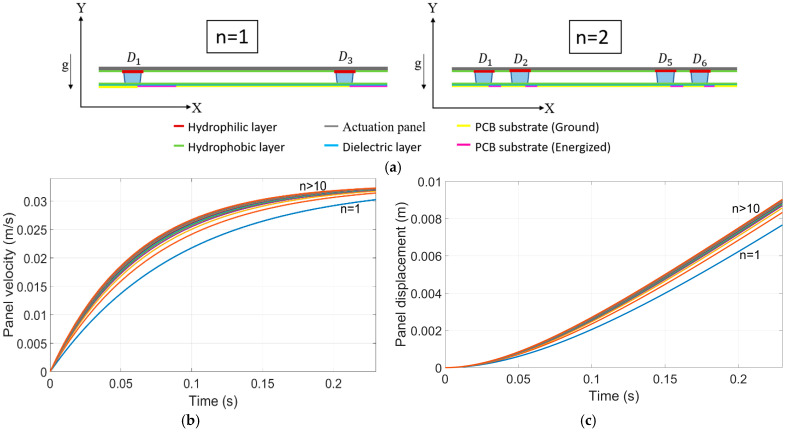
The schematic in (**a**) shows how increasing the number of droplets (*n*) enhances the electrowetting force but also increases the mass and drag on the panel. Plot (**b**) illustrates the simulation results of panel velocity. (**c**) Displacement profile of the panel for various droplet configurations (*n* = 1, 2, 3, >10) using simulation parameters from Table 1 (the electrical setup closely followed the methods section, with the key difference being that additional electrodes were actuated at precise times to maintain a constant electrowetting force on the plate, preventing it from entering a deceleration phase).

Case 1: Figure 18a shows the schematic diagram illustrating the configuration of droplets positioned on a hydrophobic layer beneath an actuation panel, driven by electrowetting forces. As the number of droplets increases, the length of the plate also increases by 1.4 L for each additional droplet (where L is the length of the droplet in the wetted section). A factor of 1.4 L is considered to prevent the merging of droplets. Additional experiments are needed to precisely determine the critical gap between two droplets as a function of droplet volume and wetting area to prevent merging. In addition, the number of active electrodes also increases, so the electrowetting force is augmented, resulting in an initial increase in the panel’s velocity. In the current simulation, each droplet has a volume of 240 μL, and with each step, n, four new droplets and four new active electrodes are added to the system. As more droplets are added, the system’s mass (both droplets and plate mass) and the corresponding drag force also increase, leading to a point where additional droplets no longer contribute to a significant rise in velocity. This effect is clearly illustrated in the velocity-time plot (Figure 18b). The simulation shows that at a specific time, such as 0.1 s for (*n* = 5) (with a total volume of 4.8 mL), the panel reaches 90% of its maximum speed while using 50% less droplets compared to (*n* = 10). Adding more droplets beyond this point increases complexity without significant performance gains. Additionally, the plot indicates that at 0.1 s, increasing the number of droplets from (*n* = 1) to (*n* = 2) results in only a slight velocity increase of less than 14%, while introducing considerable challenges, such as merging issues and fabrication difficulties. The displacement plot (Figure 18c) shows that increasing the number of droplets initially results in a steeper rise in panel displacement, as the augmented electrowetting force leads to faster acceleration. However, all configurations tend towards the same steady-state velocity, indicating that the greatest benefit of additional droplets is observed in short actuation periods, where the panel does not reach a steady state. Higher droplet counts (e.g., *n* > 10) show greater displacement than lower counts like (*n* = 1), but the differences diminish over time as the system reaches steady-state velocity. This highlights the advantage of optimizing the droplet count specifically for applications involving brief actuation periods, where quick acceleration is needed without the panel fully settling. This plateau highlights the importance of optimizing droplet count to achieve the desired displacement without unnecessarily increasing system complexity and fabrication challenges.

**Figure 19:**
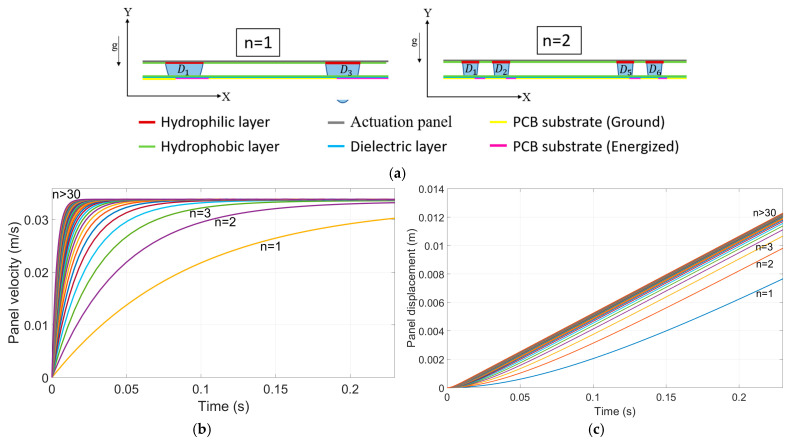
(**a**) Schematic comparison of two configurations: (*n* = 1) four single large droplets actuated by four single electrodes and (*n* = 2) multiple smaller droplets, each paired with its own electrode. In both cases, the total system volume remains constant, maintaining consistent inertia. Plot (**b**) illustrates the simulation results of panel velocity. (**c**) Displacement profile of the panel for various droplet configurations (*n* = 1, 2, 3, >30) using simulation parameters from Table 1 (the electrical setup closely followed the methods section, with the key difference being that additional electrodes were actuated at precise times to maintain a constant electrowetting force on the plate, preventing it from entering a deceleration phase).

Case 2: The velocity profile of a panel actuated by electrowetting forces was analyzed for two configurations: (1) a single large droplet with a single electrode and (2) multiple smaller droplets, each paired with its own electrode (See Figure 19a). In both cases, the total droplet volume was kept constant, ensuring the inertia remained unchanged. The length of the plate increased by 0.4 L for each additional droplet (where L is the length of the droplet in the wetted section) to prevent droplet merging. Additional experiments are needed to precisely determine the critical gap between two droplets as a function of droplet volume and wetting area to prevent merging.

In the original configuration, the panel was actuated by a single large droplet through the electrowetting force applied by a single electrode. The velocity profile demonstrates a gradual increase as the electrowetting force overcomes the system’s inertia. However, the response was limited by the single droplet and the corresponding electrode, which resulted in a lower actuation force. To improve the system’s response, the large droplet was divided into n smaller droplets. These droplets were distributed across, n, smaller electrodes, each contributing to the electrowetting force. Although the system’s total volume and inertia remained constant, the actuation force increased, allowing for faster acceleration of the panel.

In the velocity profile (Figure 19b), this modification is reflected by a steeper increase in velocity during the initial stages of motion, compared to the single-droplet configuration. The panel achieved higher velocity at a given time due to the increased force, leading to a more rapid response. The velocity continues to increase at an accelerated rate until the panel’s inertia balances the force applied. For example, at 0.1 s, increasing the number of droplets from (*n* = 1) to (*n* = 2) resulted in a 32% increase in velocity, whereas in the first configuration (case 1), the velocity increased by only 14%. The displacement (Figure 19c) reveals that as the number of droplets (*n*) increases, the panel experiences a steeper displacement over time. This behavior is attributed to the enhanced cumulative actuation force provided by multiple droplets, which accelerates the panel more effectively compared to the single large droplet configuration. As shown, when n exceeds 30, the displacement curve approaches a maximum rate, indicating that further increases in n yield diminishing returns on displacement enhancement. This outcome suggests an optimal range of droplet division that maximizes displacement while maintaining system efficiency.

To reach the same displacement, the configuration with multiple droplets (e.g., *n* = 3) achieves the target displacement more quickly than the single-droplet configuration (n = 1). In the displacement profile, with (*n* = 3), the panel reaches a given displacement in approximately 40% less time than with *n* = 1. This improvement is due to the cumulative effect of multiple droplets applying a greater actuation force, enabling the panel to accelerate and reach the target displacement more efficiently. In comparison, Case 1 only resulted in a 15% reduction in the time required to reach the same displacement. This demonstrates the effectiveness of using multiple smaller droplets in achieving faster actuation without altering the total system volume or inertia.

## 5. Conclusions and Future Work

This study has demonstrated the feasibility and effectiveness of using EWOD for actuating a rigid plate, which can be applied in applications such as microfluidic reconfigurable RF devices. The proposed EWOD system, characterized by its simple two-dimensional manufacturing process and absence of mechanical components, offers significant advantages in terms of compactness, portability, and cost-effectiveness compared to traditional micropump actuators. The experimental results validated the analytical model, with a maximum error of 7.8% between predicted and observed velocities, confirming the model’s accuracy. The study showed that the electrowetting force could propel the droplet with volume of 240 μL and panel weight of 0.92 g to a peak velocity of up to 33 mm/s at the saturation voltage, demonstrating the system’s capacity for rapid actuation. The impact of viscosity on actuation speed was also investigated, revealing that higher viscosities (e.g., 0.003 kg/m·s) significantly reduce the peak velocity due to increased resistance forces. For instance, the velocity at a viscosity of 0.001 kg/m·s was observed to be 54.5% higher compared to a fluid with a viscosity of 0.003 kg/m·s, underscoring the importance of selecting fluids with optimal viscosities for specific applications.

This study employed a 3D numerical FEM model to analyze the velocity profile and viscous dissipation force of an electrowetting droplet, revealing differences between the droplet’s center and edges that cannot be captured experimentally. The results indicated that smaller droplets exhibited faster response times and more linear behavior, whereas larger droplets showed slower velocity profile growth and non-linear characteristics, highlighting the importance of maintaining a height-to-width ratio (H/W) below 0.05 for optimal performance.

The proposed model assesses the impact of droplet number on actuation speed, particularly in scenarios like nanoliter droplet generation, where experimental validation is challenging. Two case studies demonstrate how the electrowetting actuator’s performance can be changed. In the first case, as the number of droplets increases, the actuation speed initially rises due to augmented electrowetting forces; however, additional droplets eventually lead to increased mass and drag, resulting in diminishing returns on velocity gains. For instance, increasing from one to two droplets only produces a 14% increase in velocity, while introducing significant complexities in merging and manufacturing. An alternative approach to stacking droplets can yield a 23% velocity increase without adding mass. The second case compares a single large droplet with multiple smaller droplets, revealing that the latter configuration significantly enhances actuation speed due to the increased electrowetting forces generated by multiple electrodes, achieving a 32% increase in velocity. Overall, these findings highlight the importance of optimizing droplet configurations to enhance system performance while minimizing complexity.

For future work, integrating the EWOD system with an RF device to create a closed system is recommended to address issues such as droplet evaporation. In practical applications, enclosing the droplets in a sealed cavity could effectively minimize or eliminate evaporation. Additionally, selecting a less volatile droplet fluid and filling the cavity with a fluid ambient could further reduce evaporation, enhancing system accuracy and ensuring the long-term stability of the droplets and the overall actuation system. Furthermore, investigating the effects of vibrations on the EWOD system is suggested. This research provides a foundation for further advancements in microfluidic RF devices and other electronic configurations requiring precise and reliable actuation.

## Figures and Tables

**Table 1:** Key test parameters for measuring electrowetting droplet response.

Fluid 1	Fluid 2	$\rho_{1}$	$\rho_{2}$	$\mu_{1}$	$\mu_{2}$
Water	Air	$1021\;kg/m^{3}$	$1.2\;kg/m^{3}$	0.001 kgm·s	0.000018 kgm·s
Droplet volume	γ	L	m		
240 μL	43.8 mN/m	10 mm	0.92 g		

**Table 2:** Extracted numerical coefficients.

Droplet viscous coefficients	α=0.0180
Contact-line friction coefficient	η=0.0109

## Data Availability

Data is available upon request.
